# Hesperidin from *Citrus* seed induces human hepatocellular carcinoma HepG2 cell apoptosis via both mitochondrial and death receptor pathways

**DOI:** 10.1007/s13277-015-3774-7

**Published:** 2015-07-21

**Authors:** Ratana Banjerdpongchai, Benjawan Wudtiwai, Patompong Khaw-on, Wasitta Rachakhom, Natthachai Duangnil, Prachya Kongtawelert

**Affiliations:** Department of Biochemistry, Faculty of Medicine, Chiang Mai University, Chiang Mai, 50200 Thailand

**Keywords:** *Citrus* seeds, Apoptosis, Cancer, HepG2 cells, Hesperidin, Flavonoids

## Abstract

*Citrus* seeds are full of phenolic compounds, such as flavonoids. The aims of this study were to identify the types of flavonoids in *Citrus* seed extracts, the cytotoxic effect, mode of cell death, and signaling pathway in human hepatic cancer HepG2 cells. The flavonoids contain anticancer, free radical scavenging, and antioxidant activities. Neohesperidin, hesperidin, and naringin, active flavanone glycosides, were identified in *Citrus* seed extract. The cytotoxic effect of three compounds was in a dose-dependent manner, and IC_50_ levels were determined. The sensitivity of human HepG2 cells was as follows: hesperidin > naringin > neohesperidin > naringenin. Hesperidin induced HepG2 cells to undergo apoptosis in a dose-dependent manner as evidenced by the externalization of phosphatidylserine and determined by annexin V-fluorescein isothiocyanate and propidium iodide staining using flow cytometry. Hesperidin did not induce the generation of reactive oxygen species, which was determined by using 2′,7′-dichlorohydrofluorescein diacetate and flow cytometry method. The number of hesperidin-treated HepG2 cells with the loss of mitochondrial transmembrane potential increased concentration dependently, using 3,3′-dihexyloxacarbocyanine iodide employing flow cytometry. Caspase-9, -8, and -3 activities were activated and increased in hesperidin-treated HepG2 cells. Bcl-xL protein was downregulated whereas Bax, Bak, and tBid protein levels were upregulated after treatment with hesperidin in a dose-dependent manner. In conclusion, the bioflavanone from *Citrus* seeds, hesperidin, induced human HepG2 cell apoptosis via mitochondrial pathway and death receptor pathway. *Citrus* seed flavonoids are beneficial and can be developed as anticancer drug or food supplement, which still needs further in vivo investigation in animals and human beings.

## Introduction

The incidence of liver cancer is in the first fifth ranks of mortality in the world. The risk and precipitating factors or causes include viral hepatitis B and C infection, alcoholic cirrhosis, non-alcoholic fatty liver related to obesity, prolonged consumption of drugs, or chronic hepatic exposure to xenobiotics such as aflatoxin B1 [1, 2].

Chemotherapy, immunotherapy, intravenous drug embolization, and surgery are employed to treat hepatoma cancer patients. However, the effectiveness and side effects remain a problematic issue.

Recent research on anticancer effect of natural products has been focused on cancer cell death induction. Cancer cell death induced by chemotherapy or natural products can be via apoptosis (type I programmed cell death), autophagic cell death (type II programmed cell death), and necroptosis (programmed necrosis) [3–5]. The apoptotic cells are characterized by cell membrane blebbing, cytosolic shrinkage, condensed nuclei, and apoptotic bodies with the externalization of phosphatidylserine to the outer layer of cell membrane, whereas for the autophagic cells by the generation of autophogosomes and autophagolysosomes in the cytoplasm with *Atg* gene expression and necroptotic cells by cell swelling and the loss of cell membrane integrity and inhibition by necrostatin 1 [6].

Flavonoids are abundantly found in fruits and vegetables including grains. Most *Citrus* species contains substantial amount of limonoids, flavonoids, and carotenoids in the forms of both glycoside or aglycone. The aglycone forms, naringenin and hesperetin, are the most important flavanones [7]. These phytochemical compounds contain antioxidant, antiproliferative, antiviral, antiallergic, antiinflammatory, antiatherosclerotic, and anticancer activities [8].

The structure of glycosides determines the taste of *Citrus*, for example, the compounds consist of rhamnosyl-alpha-1,2-glucose or disaccharide residue (ramnosyl-alphai-1,6 glucose) give the sweet taste, whereas rutinosides and neohesperidosides provide bitter taste [9].

Hesperidin is an active flavanone glycoside found in the *Citrus* juice, whole fruit, and peel. Some glycosids are more toxic to cancer cells, and some are less than their aglycone derivatives, depending on the types of flavonoids [10]. *Citrus* bioactive compounds also induce significantly phase II detoxifying enzyme activities, such as quinone reductase and glutathione S-transferase [11]. Flavanones, hesperidin, narirutin, and didymin, are present in orange and lemon juices [9].

*Citrus* flavonoids such as limonin and its glucosides have been reported to inhibit colon adenocarcinoma SW480 cell proliferation through apoptosis via intrinsic pathway [12]. In a comparative study of limonoid effect on cancer cell cytotoxicity, both aglycones and glycosides of limoinoids possess strong antiproliferative activity, but the latter are the apoptosis-inducing forms in neuroblastoma SH-SY5Y cell line [13].

Since, *Citrus* peel and seed are also composed of polyphenolic derivatives, such as phenolic acids and flavonoids. *Citrus* peel contains more flavonoids than the seed [14]. The *Citrus* peel and seed are by-products. It is necessary to measure their bioflavonoids’ contents and activities on cancer cell growth and the mechanism involved. There has never been any study on the amount and types of flavonoids in *Citrus* seed and the effect of *Citrus* flavonoids on human hepatic cancer cell proliferation; hence, the aims of this investigation were to identify the types of active compounds in *Citrus* seed and the inhibitory effect of *Citrus* seed flavonoids on human hepatocellular cancer HepG2 cell growth together with mechanism of cell death.

## Materials and methods

### Reagents

Bioflavonoids and ethanolic crude extract from *Citrus* seeds were obtained from Ruiheng Industry Co., Limited., Hefei City, China. Dimethyl sulfoxide (DMSO), 3-(4,5 dimethylthiazol-2-yl)-2,5-diphenyltetrazolium bromide (MTT dye), 2′,7′-dichlorohydrofluorescein diacetate (DCFH-DA), neohesperidin, hesperidin, naringin, and naringenin were obtained from Sigma-Aldrich, St. Louis, MO, USA. Dulbecco’s modified Eagle medium (DMEM), fetal bovine serum, penicillin G sodiumm and streptomycin were from GibcoBRL, Invitrogen Life Science Technologies, Thermo Fisher Scientific Inc., Waltham, MA, USA. Annexin V-Fluos kit and complete mini-protease inhibitor cocktail were obtained from Roche, Indianapolis, IN, USA. Caspase determining kits were obtained from Invitrogen Life Science Technologies, Thermo Fisher Scientific Inc., Waltham, MA, USA. Mouse monoclonal antibody to Bcl-2 associated X protein (Bax) and rabbit polyclonal to Bcl-xL, Bak, and Bid, and horseradish peroxidase (HRP)-conjugated secondary antibodies were obtained from Abcam, Cambridge, UK. Super Signal West Pico Chemiluminescent Substrate was obtained from Thermo Fisher Scientific Inc. Waltham, MA, USA.

### Characterization of active compounds in bioflavonoids from *Citrus* seeds

The commercial available bioflavonoids from *Citrus* seeds were separated through high-performance liquid chromatography by using HP (Hewlett-Packard, CA, USA) 1050 gradient liquid chromatography with DAD 1050 M coupled to a Chemstation HP and a reverse phase column Luna C18 5 μm × 150 × 2.1 mm (Phenomenex, Torrance, CA, USA). The running solvents were as follows: 0.1 % formic acid in water and 0.1 % formic acid in acetonitrile. Gradients were as follows: 0–35 min 6–50 %, 35–45 min 50–100 % and then back to 6 %. The column was equilibrated for 15 min prior to analysis. The flow rate was 0.4 ml/min, injection volume was 10 μl, and column temperature was at 40 °C. The UV-Vis spectra were recorded from 280 to 400 nm, with detection at 280 and 365 nm.

### Cell culture

Human hepatocellular carcinoma HepG2 cell line was kindly given by Associate Professor Dr. Prachya Kongtawelert, Excellence Center of Tissue Engineering and Stem Cells, Department of Biochemistry, Faculty of Medicine, Chiang Mai University. HepG2 cells were cultured in DMEM medium supplemented with 10 % fetal bovine serum with 25 mM sodium bicarbonate, 20 mM HEPES, 100 U/ml penicillin G sodium, and 100 μg/ml streptomycin at 37 °C and 5 % CO_2_.

### MTT assay

The HepG2 cells were treated with crude ethanolic extract from *Citrus* seeds, bioflavonoids, and commercially available active compounds, viz., neohesperidin, hesperidin, naringin, and naringenin at various concentrations for 24 h. Then, MTT dye (dissolved in DMSO less than 0.1 %) was added at final concentrations of 100 μg/ml and incubated for 4 h at 37 °C in CO_2_ incubator. The medium was removed, and the violet crystal was dissolved in DMSO for 30 min. The absorbance was determined by using spectrophotometer at 540 nm and reference wavelength at 630 nm (BioTek, Winooski, VT, USA). The concentrations at 10, 20, and 50 % inhibitory effect on proliferation of the extract, bioflavonoid, and each active compound on human hepatocellular carcinoma cells were determined by plotting the graph of cell viability and concentrations of the natural products [15].

### Annexin V-FITC and PI staining and flow cytometry

HepG2 cells were treated with hesperidin at IC_0_, IC_10_, IC_20_, and IC_50_ for 24 h and then stained with annexin V-fluorescein isothiocyanate (FITC) and propidium iodide (PI) for 15 min. The cells were washed with phosphate-buffered saline and resuspended in annexin V-FICTC and PI as indicated in the annexin Fluos kit. The stained cells were measured by using flow cytometer and analyzed with Cell Quest (software program) of flow cytometer (Becton Dickinson, Frankin Lakes, NJ, USA) [16].

### Determination of caspase-9, -8, and -3 activities

After, HepG2 cells were treated with hesperidin at various concentrations for 24 h. The cells were harvested, and cell lysate was incubated with caspase-specific tetrapeptide substrates, which were labeled with *p*-nitroaniline. For caspase-9, LEHD-*p*NA, caspase-8, IETD-*p*NA and caspase-3, DEVD-*p*NA were used accordingly for measuring the activities of the corresponding caspases as described in each kit. The lysate was incubated with each substrate for 1 h, and then, the absorbance was determined by spectrophotometry technique (using microplate reader, BioTek, Winooski, VT, USA) [17].

### MTP assay by flow cytometry

Twenty-four-hour hesperidin-treated hepatoma HepG2 cells were incubated with dihexyloxacarbocyanine iodide (DiOC_6_) at 40 nM (as final concentration) for 15 min and determined for the loss of mitochondrial transmembrane potential (MTP) by using fluorescence activated cell sorter (FACS) (Becton Dickinson, Frankin Lakes, NJ, USA) [3].

### Determination of ROS generation

After, the hepatic cancer HepG2 cells were incubated with hesperidin at various concentrations for 4 h. DCFH-DA was added to the final concentration of 5 μM and incubated for 15 min before determining the fluorescence intensity of DCF employing flow cytometer (Becton Dickinson, Frankin Lakes, NJ, USA) [18].

### Determination of proteins involved in apoptosis pathway by Western blot

HepG2 cells were treated with hesperidin at various concentrations for 24 h and washed once in ice-cold PBS. The treated cells were incubated at 4 °C for 10 min with ice-cold cell lysis buffer (250 mM sucrose, 70 mM KCl, 0.25 % Triton X-100 in phosphate-buffered saline containing complete mini-protease inhibitor cocktail). The cell lysate was centrifuged at 20,000 *× g* for 20 min; the supernatant (50 μg, determined by Bradford method) was separated by 15 % SDS-PAGE and transferred onto nitrocellulose membrane. After treating with 5 % non-fat milk in PBS containing 0.2 % Tween-20, membrane was incubated with mouse monoclonal antibody to Bax and rabbit polyclonal to Bcl-xL, Bak, and Bid, followed by appropriate horseradish peroxidase (HRP)-conjugated secondary antibodies (1:20,000). Protein bands were visualized on X-ray film with Super Signal West Pico Chemiluminescent Substrate. The band density was determined by using densitometer compared to the control protein actin [4].

### Statistical analysis

The data were demonstrated as mean ± S.D. performed in triplicate and three times independently. The statistical difference was determined by using one-way ANOVA, and two groups of data were compared by Student’s *t* test with the *p* value of <0.05 by using SPSS program version 16.0.

## Results

### Active compounds in *Citrus* seed are neohesperidin, naringin, and hesperidin

**Fig. 1:**
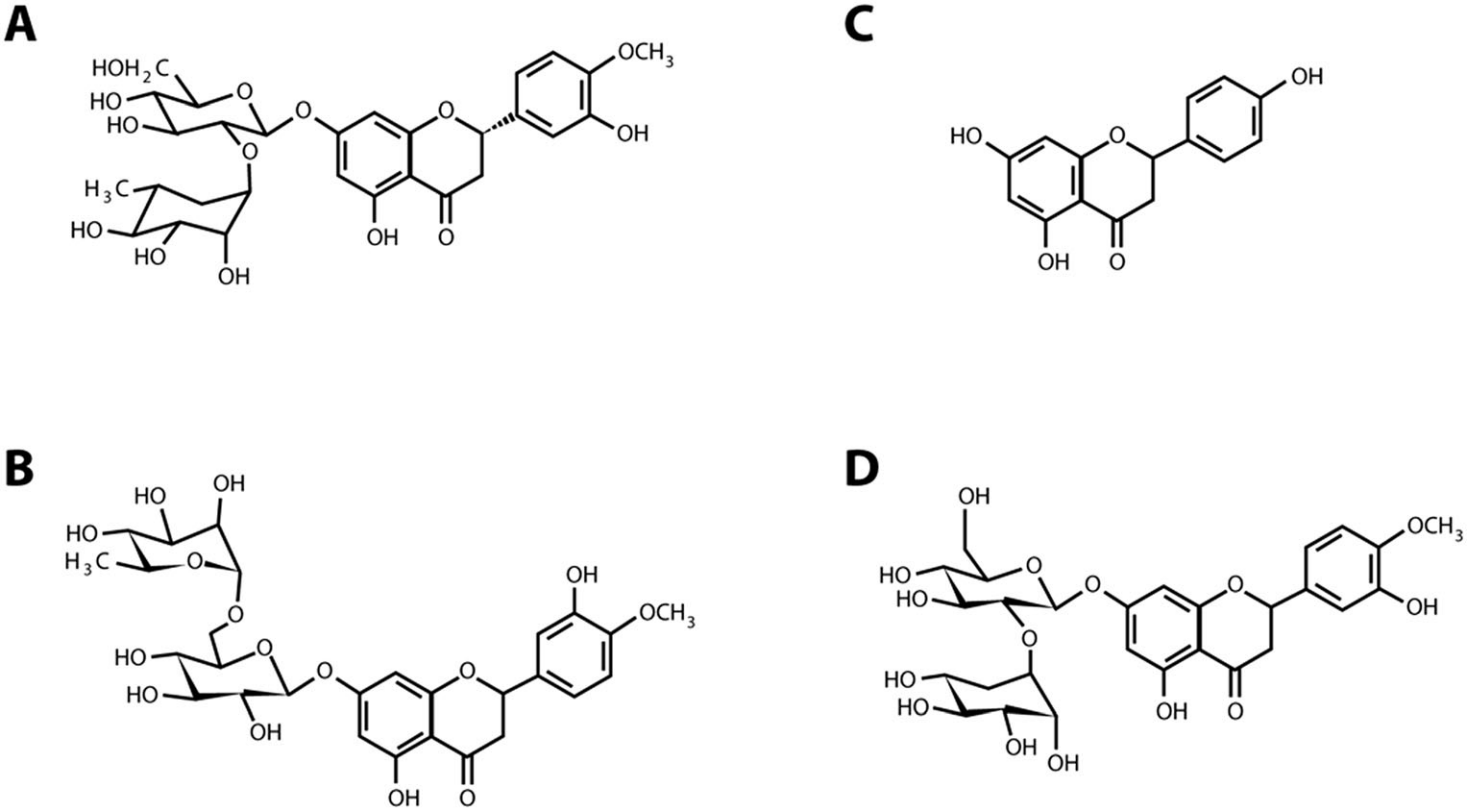
Chemical structure of active phytochemicals found in *Citrus* seed: **a** neohesperidin, **b** hesperidin, **c** naringenin, and **d** naringin

**Fig. 2:**
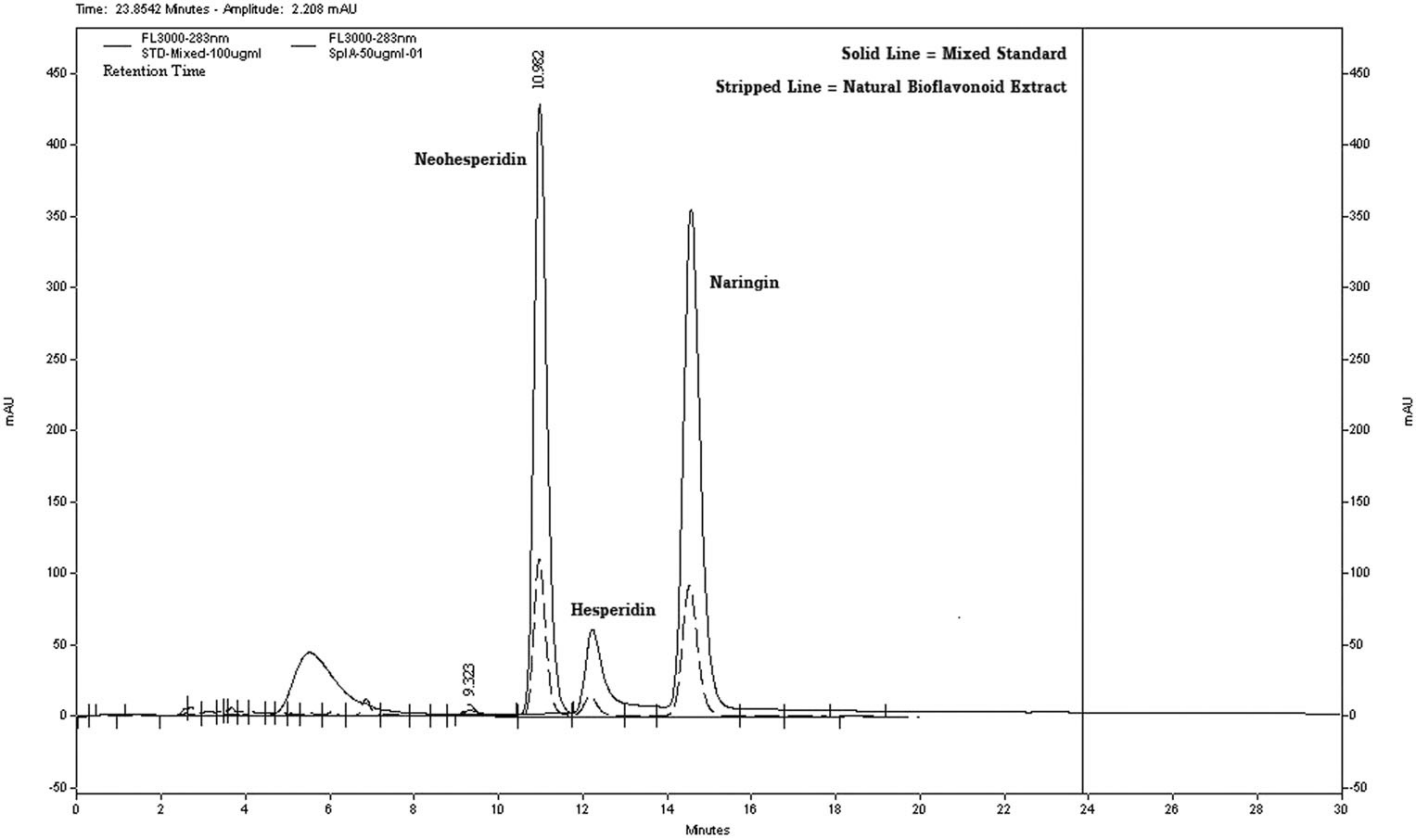
Chromatogram of the active compounds separated by high-performance liquid chromatography (HPLC) technique. The active standard phytochemicals of the flavonoids (*continuous line*) were compared to the compounds found in bioflavonoid from *Citrus* seed (*striped line*), viz., neohesperidin, hesperidin, and naringin

Bioflavonoids from *Citrus* seed composed of several active phytochemicals, such as neohesperidin, naringin, and hesperidin. The structures of active compounds are shown in Fig. 1, viz. neohesperidin (1a), hesperidin (1b), aglycone naringenin (1c) and compared to the glycoside form of naringenin, naringin (1d). Each compound peak in the extract (striped line) was compared to the standard compound, shown in solid line (Fig. 2). The standard and the active phytochemicals in *Citrus* seed superimposed to each other; the result was comparable to the flavanones [19]. The yields of neohesperidin, hesperidin, and naringin, identified in *Citrus* seed extract, were 17.11, 7.69, and 20.30 % (*w*/*w*), respectively. The structures of neohesperidin and hesperidin are isomers.

### Cytotoxic effect of *Citrus* extract, bioflavonoids, and active compounds on human HepG2 cells

**Fig. 3:**
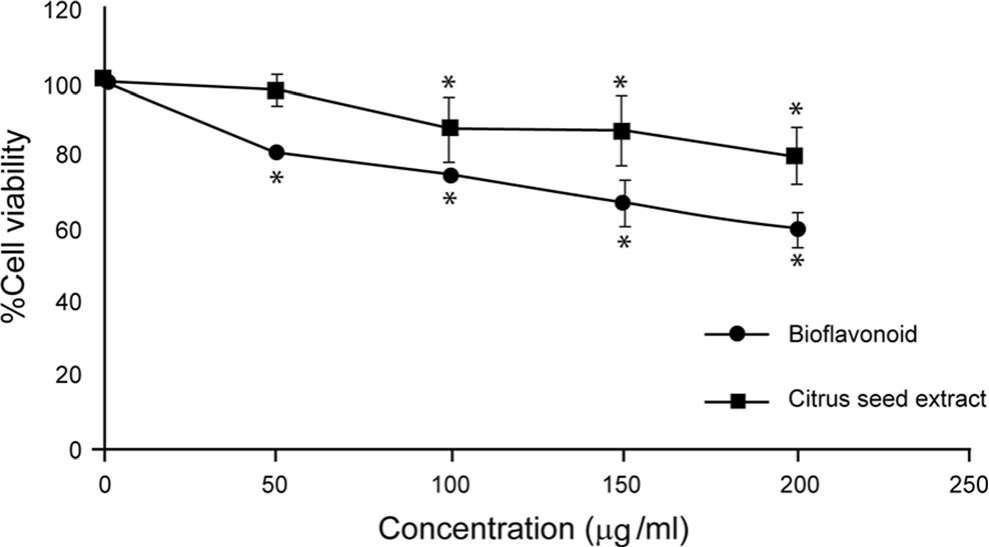
Cell viability of human hepatocellular carcinoma HepG2 cell treated with crude ethanolic extract and bioflavonoids from *Citrus* seed. The significance of statistical value compared to control (without treatment) is marked with *asterisk*, *p* < 0.05

Both ethanolic crude extract of *Citrus* seed and bioflavonoids from *Citrus* seed slightly inhibited HepG2 cell proliferation with the IC_50_ values of more than 200 mg/μl as shown in Fig. 3. Bioflavonoids were toxic to HepG2 cells more than the crude extract of *Citrus* seed.

**Fig. 4:**
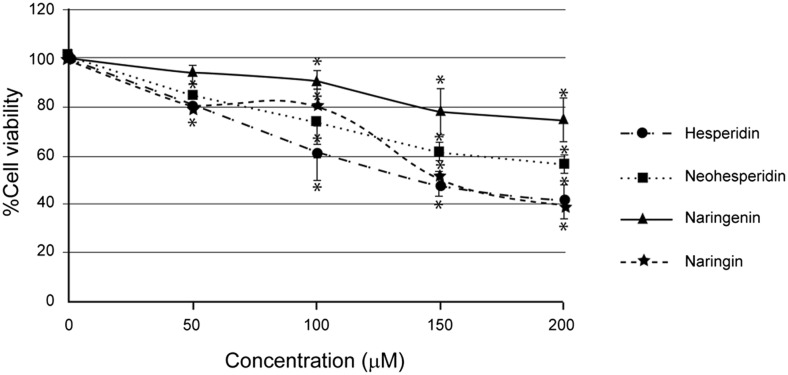
Cell cytotoxicity of human hepatocellular carcinoma HepG2 cells treated with phytochemical neohesperidin, hesperidin naringin, and naringenin. HepG2 cells were treated with various concentrations of the active compounds from *Citrus* seed for 24 h, and the cell viability was measured by MTT assay as described in “Materials and methods.” The significance of statistical value compared to control (without treatment) is marked with *asterisk*, *p* < 0.05

Hesperidin was toxic to human hepatocellular carcinoma HepG2 cells, with the IC_10_, IC_20_, and IC_50_ levels of 23.65 ± 2.75, 49.06 ± 4.06, and 150.43 ± 12.32 μM, respectively. When compared to the other two bioflavonoids found in *Citrus* seeds, neohesperidin and naringin, both were less toxic to HepG2 cells with IC_50_ levels more than 200 and 172.00 ± 10.39 μM, respectively, as shown in Fig. 4.

From the present data, flavonoids from *Citrus* seed inhibited human hepatic cancer HepG2 cell growth with the sensitivity as follows: hesperidin > naringin > neohesperidin > naringenin (Fig. 4). The cytotoxicity of naringin in its glycoside form was compared with aglycone naringenin. Glycoside form was more toxic to hepatic cancer cells than aglycone form in this HepG2 cell model. The most toxic compound for HepG2 cell line was hesperidin; hence, hesperidin was selected for further study of the mechanism of cell death.

### Hesperidin-induced apoptosis in HepG2 cells

**Fig. 5:**
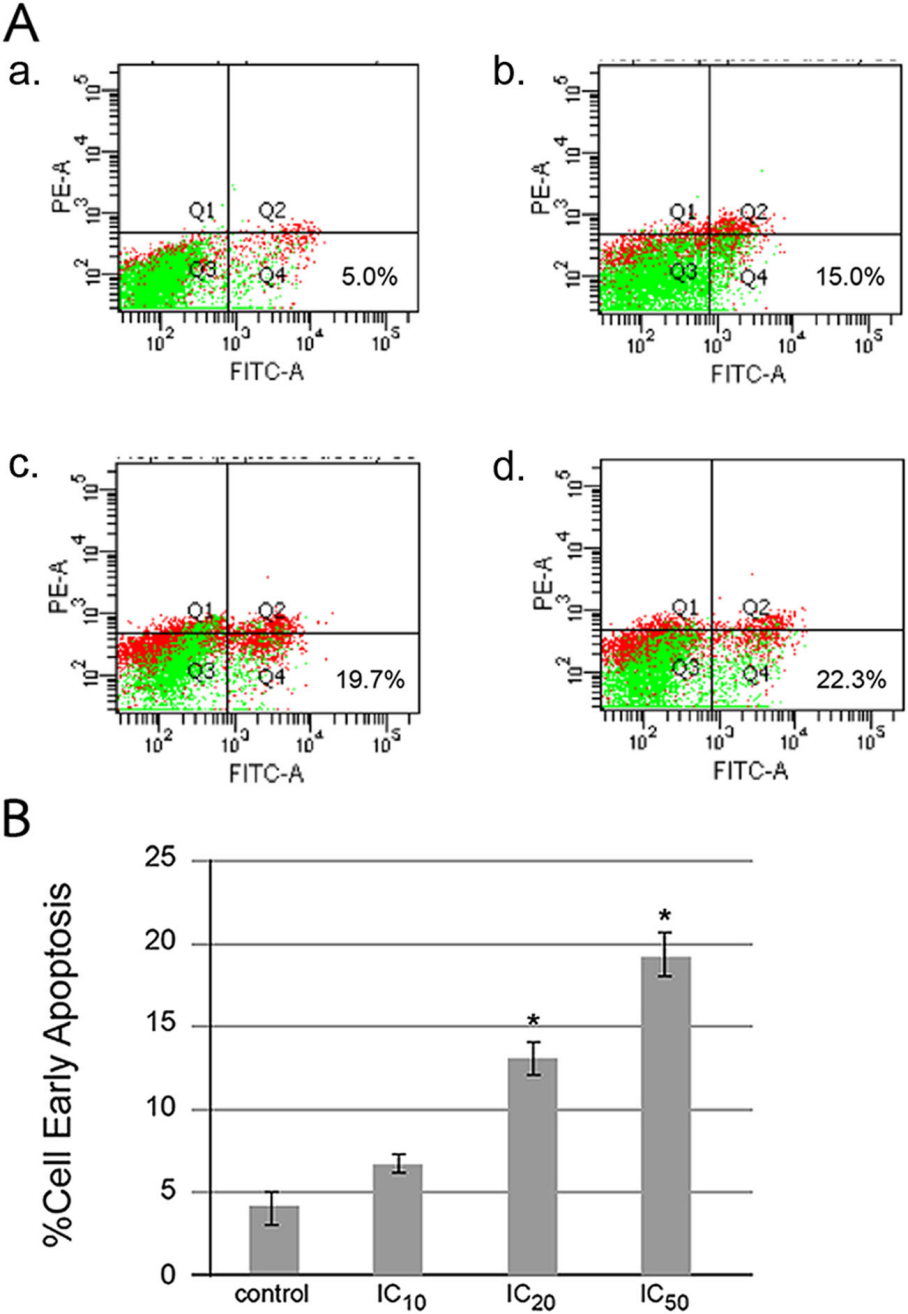
Apoptotic cell death induced by hesperidin. The apoptotic HepG2 cells were stained with annexin V-fluorescein isothiocyanate and propidium iodide and employing flow cytometry technique as described in “Materials and methods.” The *dot plots* (**a**) of cells in each concentration of hesperidin are (*a*) control (without hesperidin treatment) and various concentrations of hesperidin at (*b*) IC_10_, (*c*) IC_20_, and (*d*) IC_50_ in μM unit. *Bar graphs* (**b**) of the percentage of early apoptotic cells in each concentration are shown. The significance of statistical value compared to control (without treatment) is marked with *asterisk*, *p* < 0.05

Determination of phosphatidylserine (PS) externalization is used to determine apoptotic or necrotic cell death by flow cytometry technique [20]. HepG2 cells treated with hesperidin at IC_10_, IC_20_, and IC_50_ concentrations showed the characteristics of apoptosis, i.e., the translocation of phosphatidylserine (PS) to the outer layer of cell membrane demonstrated by annexin V-fluorescein isothiocyanate (FITC) and propidium iodide (PI) staining at PS and DNA (nucleus), respectively, employing flow cytometry technique as shown in Fig. 5a, b. Percentage of cells in right lower quadrant (early apoptotic cells) was higher in a dose-response manner as shown in dot plot (Fig. 5a) and bar graphs (5b). The flip-flop of PS to the outer layer of cell membrane is a unique character of early apoptotic cells.

### Activation of caspase-9, -8, and -3 activities in hesperidin-induced HepG2 cell apoptosis

**Fig. 6:**
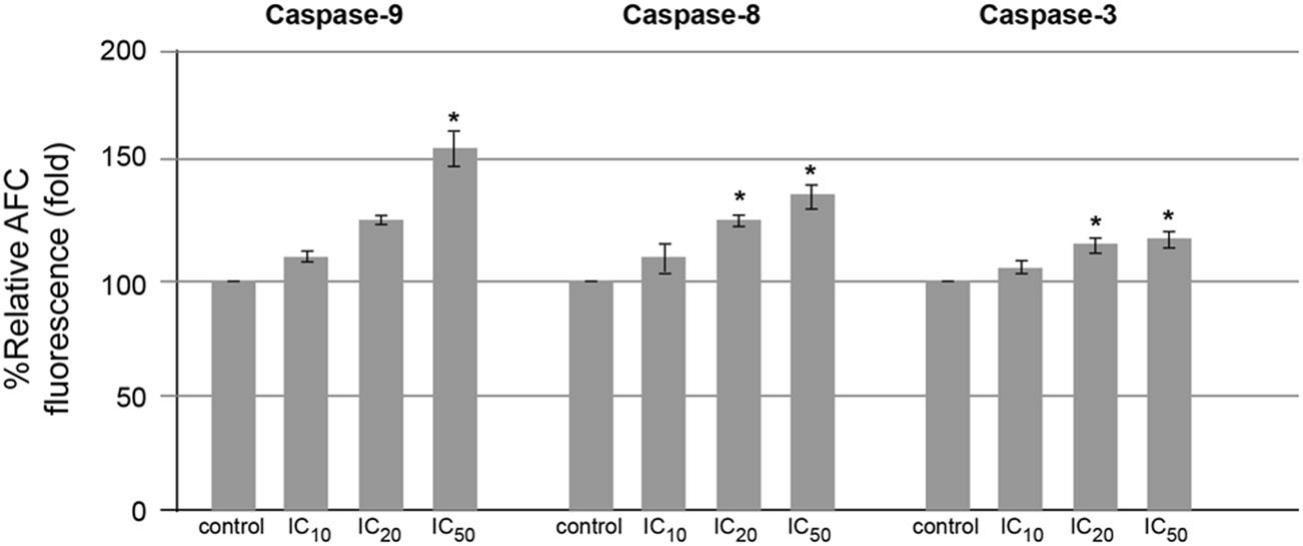
Caspase activities of hesperidin-treated HepG2 cells. The caspase-9, -8, and -3 activities of HepG2 cells treated with hesperidin for 24 h were measured by using specific tetrapeptide substrates tagged with para-nitroaniline, and the absorbance of cleaved *p*-NA was determined by spectrophotometer. The significance of statistical value compared to control (without treatment) is marked with *asterisk*, *p* < 0.05

Eupatorin, a naturally occurring flavone, inhibits cell proliferation in human leukemic HL-60, U937, and Molt-3 cells via inducing cell cycle arrest at G2-M phase and induces apoptotic cell death involving activation of multiple caspases via caspase-9, -8, -6, and -3/7 [21]. Therefore, HepG2 cells were treated with hesperidin at various concentrations for 24 h, and the cells were investigated for caspase-9, -8, and -3 activities by using specific tetrapeptide substrates labeled with colorimetric substrates. It was found that para-nitroaniline labeling to Leu-Glu-His-Asp (LEHD-*p*NA), which was specific for caspase-9; or Iso-Glu-Thr-Asp-*p*NA for caspase-8; or Asp-Glu-Val-Asp (DEVD-*p*NA) for caspase-3/7, respectively, is measured for the absorbance by spectrophotometer. When the tetrapeptide was cleaved accordingly after aspartate residue in each tetrapeptide, the *p*-NA is freely released and it absorbs the visible light at 410 nm. It was found that caspase-9, -8, and -3 activities increased in hesperidin-treated HepG2 cells as shown in Fig. 6.

### Loss of MTP in hesperidin-treated HepG2 cells

**Fig. 7:**
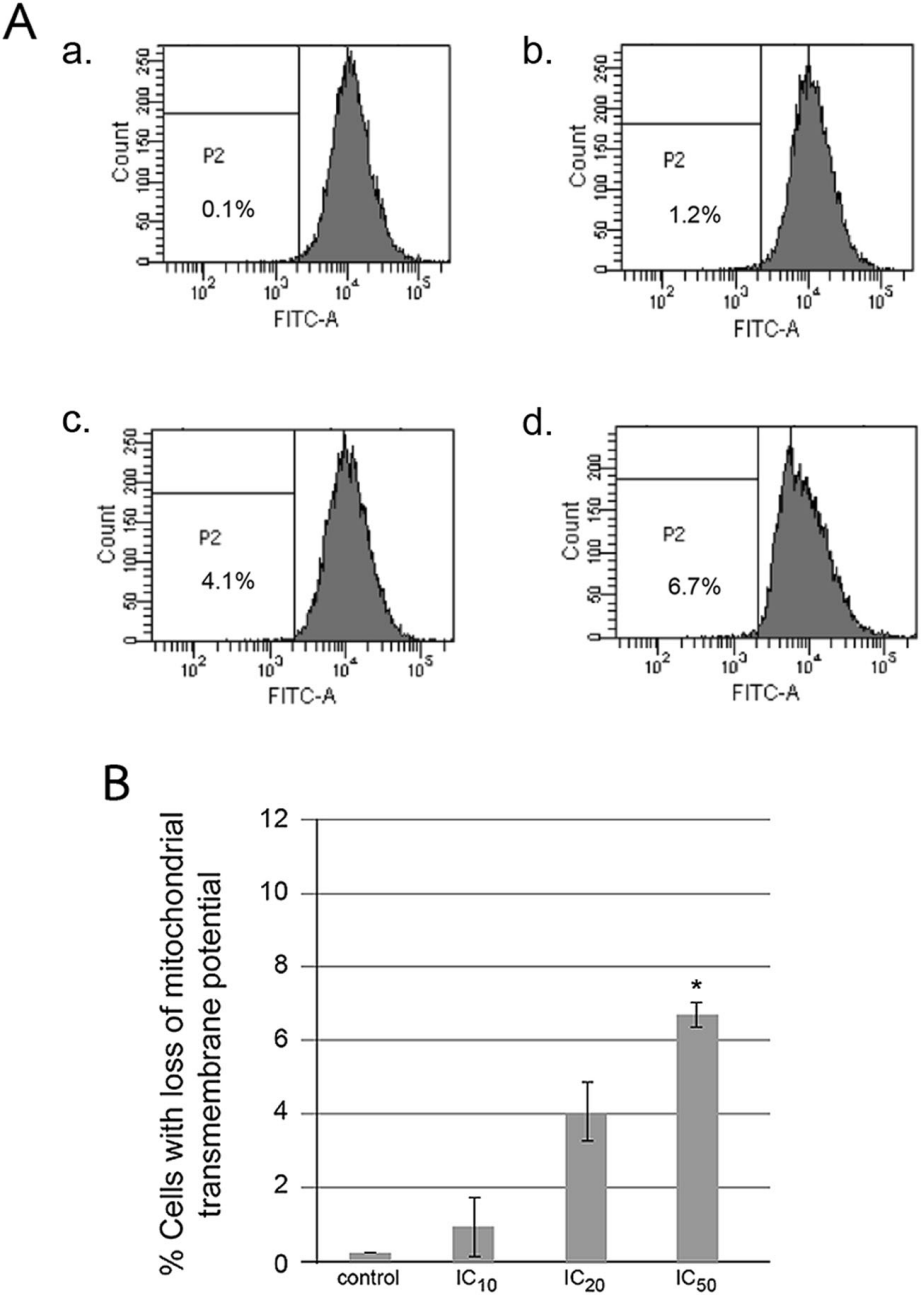
Reduction of mitochondrial transmembrane potential (MTP) of HepG2 cells after treatment with hesperidin. The human hepatic cancer cells were treated with hesperidin at IC_0_, IC_10_, IC_20_, and IC_50_ concentrations for 24 h, and MTP was measured by staining with DiOC_6_ employing flow cytometry. The relative fluorescence intensity was measured. The histograms (**a**) and bar graphs (**b**) of percentage of cells with loss of MTP are shown. The significance of statistical value compared to control (without treatment) is marked with *asterisk*, *p* < 0.05

Mitochondrial pathway involves the reduction of transmembrane potential of apoptotic cancer cells induced by stigmalactam, a natural product in an aristolactam type alkaloid group [22]. In 24-h hesperidin-incubated HepG2 cells, the changes of MTP were determined by using ionic fluorochrome, 3,3′-dihexyloxacarbocyanine iodide (DiOC_6_), which is up taken by the mitochondria. The relative DiOC_6_ fluorescence level is high in normal cells. But in apoptotic cells, the mitochondrial transmembrane permeability is lost and allowed the dye to leak into cytoplasm leading to less fluorescence intensity (Fig. 7a). Hesperidin caused the reduction of MTP, and percentage of cells with decreased fluorescence intensity was significantly increased dose dependently as shown in Fig. 7b.

### The absence of reactive oxygen species generation in hesperidin-treated HepG2 cells

**Fig. 8:**
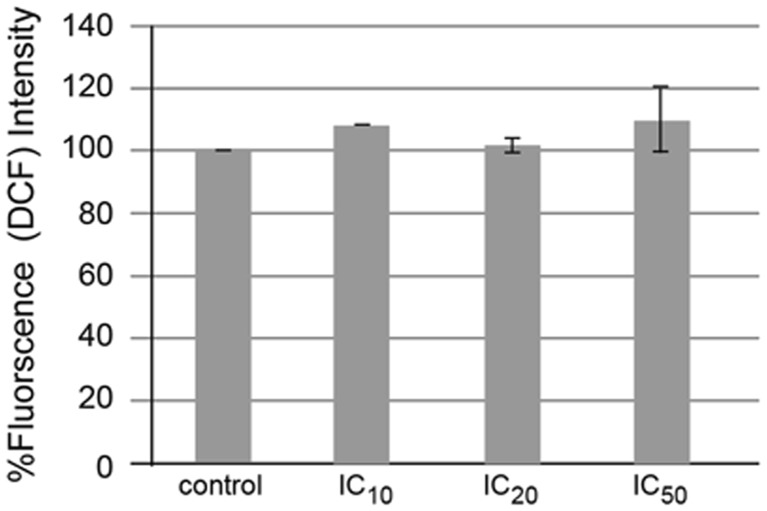
ROS generation by HepG2 cells after treatment with hesperidin. The cells were treated with hesperidin at various concentrations for 4 h and stained with DCFH-DA. The fluorescence intensity was measured compared to control without treatment

Apoptosis pathway via intrinsic and extrinsic pathways can induce the ROS generation via mitochondria and blocking NF-kappaB transcription factor in non-small cell lung cancer A549 cells induced by a histone deacetylase (HDAC) inhibitor, romidepsin [23, 24]. Human hepatocellular carcinoma HepG2 cells were treated with hesperidin for 4 h and stained with 2’,7’-dichlorohydrofluorescein diacetate (DCFH-DA) and using flow cytometer to detect the fluorescence intensity of 2′,7′-dichlorofluorescein (DCF). The ester bond in DCFH-DA is cleaved by esterase in cytoplasm to obtain DCFH, and then, DCFH is oxidized by peroxide radicals or hydrogen peroxide to yield DCF giving green fluorescence. By employing flow cytometry technique, the relative fluorescence intensity was compared with that of control (without treatment). There was no statistically significant alteration of the ROS generation when compared to the control (without hesperidin treatment), *p* value > 0.05 (Fig. 8).

### Increased levels of proapoptotic Bcl-2 family proteins and a decrease in antiapoptotic Bcl-xL protein

**Fig. 9:**
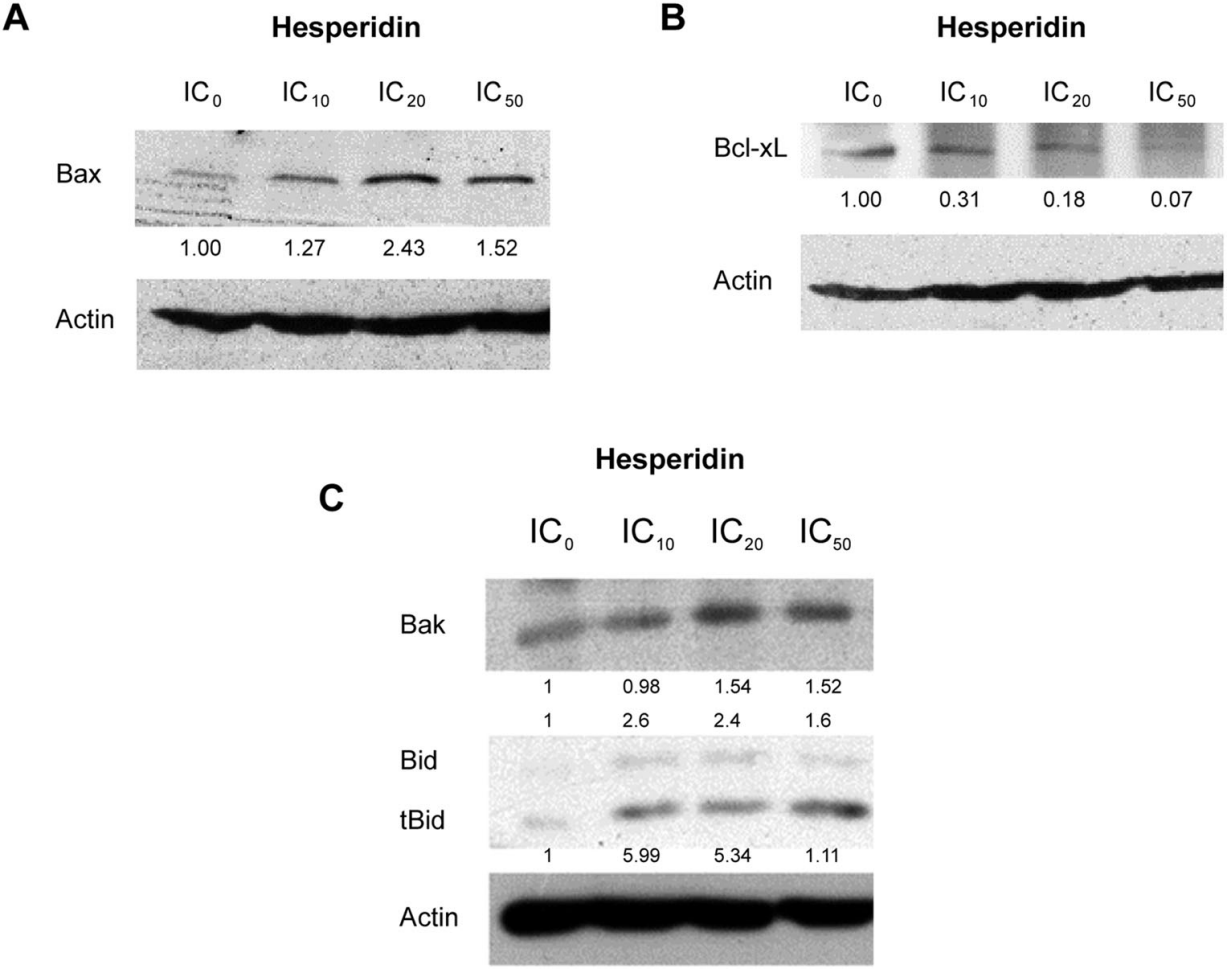
Apoptotic Bcl-2 family protein expression levels in hesperidin-treated HepG2 cells. HepG2 cells were treated with hesperidin at various concentrations for 24 h. The expression levels of proapoptotic Bax (**a**), antiapoptotic Bcl-xL (**b**) and proapoptotic Bak and BH3-only Bcl-2 family protein Bid together with truncated form (tBid) (**c**) were demonstrated by Western blot. The *number* under the bands was the fold (s) compared to control as verified by using actin as a constitutive protein, whereas the *number* above Bid bands was its expression levels compared to actin

The B-cell lymphoma 2 (Bcl-2)/Bax ratio is used as a measure of vulnerability to apoptosis [25]. The Bcl-xL/Bax was applied in the same aspect for determining the apoptosis of HepG2 cells when incubated with hesperidin, a flavonoid, found in *Citrus* seeds. Protein expression levels of the Bax (Fig. 9a), Bcl-xL (Fig. 9b), and Bak, Bid, and tBid proteins (Fig. 9c) were measured by using immunoblot. The expression of proapoptotic Bax, Bak, and tBid proteins was higher whereas the Bcl-xL and proform of Bid protein levels were lower (compared to untreated condition) in a dose-response manner (Fig. 9). Truncated Bid (tBid) is a marker of caspase-8 activity in proteolysis of Bid, a BH3-only protein in Bcl-2 family.

## Discussion

*Citrus* seed contains valuable bioflavonoids with the bioactivities on apoptosis induction in human hepatocellular cancer cells in an in vitro model. The source of *Citrus* bioflavonoid in the present experiment was from the Chinese Company. The active flavanone glycosides were neohesperidin, hesperidin, and naringin, of which the structures are shown in Fig. 1 and their HPLC chromatogram in Fig. 2. Even though the composition of *Citrus* flavonoids in different species and cultivars of *Citrus* fruits grown in different areas produces different types of flavonoids, the present report of three flavanone glycosides corresponded to the same three flavanones found in *Citrus* juice, or whole fresh fruit, or pulp, or peel, or albedo and *Citrus* flavonoid contents in traditional Chinese medicinal (TCM) food ingredients of Eastern China, such as from *Citrus aurantium* L. [19, 26]. The structure of these three compounds are identified by employing high-performance liquid chromatography (HPLC) and gas chromatography-mass spectrometry (GC-MS) techniques [26].

The cytotoxic effect of hesperidin was highest (Fig. 4), and this phytochemical was selected for further experiments to identify mode and mechanism of cell death. Hesperidin can induce both apoptotic and autophagic cell death in colon carcinogenesis [27]. There are also reports demonstrating that green tea polyphenols [5] and shikonin [28] can induce cancer cell death via necroptosis, which is a regulated or programmed necrosis. Therefore, the mode of cell death induced by *Citrus* flavanone, hesperidin, was identified in hepatic cancer cells. It was found that the HepG2 cell death was early apoptosis in a dose-response manner because of the externalization of phosphatidylserine without propidium iodide staining to the DNA, which was detected by annexin V-FITC/PI and flow cytometry (Fig. 5a, b).

Since the apoptotic cell death can be activated through intrinsic (mitochondrial) and/or extrinsic (death receptor) pathways. The initiator caspase-9 involves mitochondrial pathway whereas caspase-8 is mediated in death receptor pathway. Caspase-3/6/7 are effector or executioner caspases, and they are activated in both pathways, and these caspase activities were measured to confirm the apoptotic cell death [29]. The molecular targets of hesperidin are caspases, Bcl-2, and Bax for the induction of apoptosis and cyclooxygenase-2 (COX-2), matrix metalloproteinase-2 (MMP-2), and MMP-9 for the inhibition of angiogenesis and metastasis [30]. Hesperidin induces human colon cancer SNU-C4 cell apoptosis as determined by a decrease in messenger RNA (mRNA) expression of *bcl-2* and an increase of *bax* mRNA levels with an increase of caspase-3 expression and activity [31]. Hence, the caspase-9, -8, and -3 activities and the Bcl-2 family protein levels were determined. The caspase-9, -8, and -3 activities were significantly induced and increased in hesperidin-treated cells in a dose-response manner and especially at IC_50_ level (Fig. 6). Percentage of HepG2 cells with the loss of MTP was significantly increased at IC_50_ level whereas the mean fluorescence intensity of DiOC_6_ decreased. This suggested the involvement of mitochondrial dysfunction (Fig. 7).

ROS levels in the HepG2 cells after treatment with hesperidin did not change (Fig. 8) even though the increased ROS production is evidenced in some natural product-inducing cancer cell apoptosis [3]. This might be as hesperidin did not induce apoptosis via oxidative stress or/and its antioxidant/prooxidant activities are balanced resulting in an unchanging of the DCF fluorescence intensity in the hesperidin-treated cells. Nevertheless, the ROS may not be the peroxide species since DCFH detects only hydrogen peroxide or peroxide radicals. Other ROS such as superoxide anion radicals, hydroxyl radicals, or reactive nitrogen species (RNS) including peroxynitrite radicals may be produced.

The levels of proapoptotic proteins (Bax, Bak, and tBid) were increased whereas Bcl-xL protein expression was reduced (Fig. 9), which confirmed the involvement of mitochondrial pathway. Bax and Bak translocate from cytoplasm to mitochondrial outer membrane forming channel for protein release from mitochondrial intermembranous space, such as cytochrome c, Smac/Diablo, and apoptosis inducing factor (AIF) [32] to mediate apoptosis characters, which are formation of apoptosome, DNA degradation, and activation of caspase-9 and 3. Caspase-8-activated proteolysis of Bid can be mediated from the death receptor to mitochondrial dysfunction pathway as demonstrated by an increase in truncated Bid (tBid) level (Fig. 9). This confirmed the mechanism of apoptotic cell death to be via both mitochondrial and death receptor apoptotic pathways.

The anticancer potential of hesperidin is demonstrated by inhibiting cancer cell growth and proliferation via apoptosis induction [30]. The *Citrus* hesperidin is also reported to induce apoptosis in human hematopoietic pre-B NALM-6 leukemic cells by inducing p53 and inhibiting NF-kappaB signaling pathways [33].

In addition, hesperidin can inhibit human Burkitt’s lymphoma Ramos cell proliferation and induce apoptosis by abrogating the constitutive and doxorubicin (Dox)-induced NF-kappaB activation [34]. The potential strategy using *Citrus* bioflavonoids with the conventional heterocyclic drugs has been proposed to treat invasive brain cancer (gliomas) [35]. Hesperidin increases cytotoxicity of Dox on human cervical cancer HeLa cells [36], but in Dox-sensitive cancer cells, there is no synergistic or additive effect of hesperidin on apoptotic induction [37]. This phytochemical can attenuate cisplatin-induced acute renal injury by reducing oxidative stress, inflammation, and DNA damage, which are the adverse effects of this drug through ROS production and apoptosis in rat kidneys. Therefore, the benefit of combining hesperidin with other chemotherapeutic agents remains controversial, which may depend on the types of drugs and cancer cells.

There are many reports of clinical targeted strategy against Bcl-2 family proteins for cancer therapy. A small-molecule Bcl-2-BH4 antagonist is highly selective to the antiapoptotic Bcl-2 family proteins with specific binding affinity, changing their conformation and converting the antiapoptotic proteins to cell death inducers. The compound suppresses the growth of human cancer xenografts in mice [38]. Another type of small-molecule, Bax agonist, also induces conformational alteration in Bax protein by blocking serine 184 phosphorylation, enhancing the formation of Bax oligomers to become the channels at mitochondrial outer membrane leading to cytochrome c release and apoptosis induction in human lung cancer cells [39].

Niclosamide (C_13_H_8_C_l2_N_2_O_4_) is also a small-molecule drug with teniacidal anthelmintic activity against tapeworms; the mechanism of death induction is via uncoupling oxidative phosphorylation in the parasite. It is a potent STAT3 inhibitor, which interferes with STAT3 transcriptional activity via inhibition of its phosphorylation and nuclear translocation. Bcl-2, Bcl-xL, and Mcl-1, antiapoptotic Bcl-2 family proteins, are highly expressed in human cancer through the enhanced binding of transcription factor STAT3 dimer at their promoter regions. STAT3 homodimer is formed by the phosphorylation of its Tyr705 by the activated Janus-associated kinase (JAK) or Src. Therefore, STAT3 is an intriguing target molecule for cancer treatment by suppressing antiapoptotic Bcl-2 family gene transcription through inhibition of JAK/STAT3/Bcl-2/Bcl-xL survival pathway employing niclosamide. The drug reverses the radioresistance of human lung cancer to improve the efficiency and efficacy of response in the patients with previous radiotherapy resistance [40].

Since hesperidin regulated various Bcl-2 family protein expressions (Fig. 9), including Bcl-xL (a BH4-domain-containing Bcl-2 family protein) and Bax, the targeting and/or co-targeting of these proteins by the small molecules and hesperidin is (are) considered to be a promising strategy for hepatic cancer therapy.

The glycoside forms have commercial potential since it contains sweet taste. The properties of cellular permeability, water solubility, and metabolic change all play pivotal roles in the efficacy of natural phytochemical performance in human beings, which should be concerned to achieve the sufficient amount of active form [41]. The cytotoxic effect against HepG2 cells of naringenin (aglycone) was less than naringin (glycoside form) by MTT assay (Fig. 4). The structural change of the compounds at the molecular level after metabolism or digestion affects their functions or bioactivities at cellular level and in animal or human being model. Hesperetin, an aglycone form or an in vivo metabolite of hesperidin, also contains the anticancer and cardiovascular disease prevention and therapy [30].

Although hesperidin is not widely used as a therapeutic drug by itself, it has been in the list of Martindale Extra Pharmacopoeia to be an ingredient of various recipes internationally used for cardiovascular disorders [42]. The roles of hesperidin for treatment of other diseases are in recent clinical trials of applying hesperidin and its derivatives in patients with cardiovascular diseases, including heart failure, atherosclerosis, myocardial infarction, and hypertension. The valuable effects of hesperidin in the treatment of diabetes and dyslipidemia with its antiplatelet and anticoagulant effects are also investigated [30]. Its estrogenic effect, such as decreasing serum and hepatic lipid and reducing osteoporosis in ovariectomized rats, is documented [36]. Hence, hesperidin may be used as a phytoestrogen supplement in menopausal individuals.

The effects of hesperidin on normal cells are reported, such as hesperidin can protect normal renal cells from oxidative injury [43]. It prevents quail neural crest cells from aflatoxin B1 toxicity [44]. Furthermore, the neuroprotective effects of hesperidin in Alzheimer’s disease and Parkinson’s disease models induced by aluminum chloride [45] and rotenone [46], respectively, are also evidenced. In rats, the chemoprevention of hesperidin in azoxymethane-induced colon carcinogenesis [47] also established. This increases the value of the compound in *Citrus* by-product, i.e., the seed. Hesperidin has a high potential to be applied for chemoprevention and clinical trials as an anticancer agent.

## Conclusion

Neohesperidin, hesperidin, and naringin are active bioflavonoids found in *Citrus* seed. Hesperidin induced human hepatocellular carcinoma HepG2 cell apoptosis via both intrinsic and extrinsic pathways. Further, an in vivo model research is required before using hesperidin in the clinical aspects.
